# Hospital and Institutional News

**Published:** 1916-05-13

**Authors:** 


### HOSPITAL AND INSTITUTIONAL NEWS.

### NEWSPAPERS AND THE WAR.

The Hospital, like many another newspaper, will have to rely upon the support of its readers to meet the enormous increase in the cost of production due to the war. During the last few months, owing to the restrictions on the import of pulp, heavy freightage, and other causes, the price of paper has increased 100 per cent., and there is no sign that this tendency to increase has yet attained a maximum. Paper is obviously a main item of expenditure in newspaper production, and we have confidence that our readers will co-operate and help to meet the present crisis, should it be found necessary to increase the price of The Hospital for the period of the war.

### FLORENCE NIGHTINGALE'S OPINION OF SHAKESPEARE.

The subject of Shakespeare on Nursing has not received so much attention as that of his medical knowledge; but Florence Nightingale regarded him as an authority, as the following extract from Sir Edward Cook's invaluable Life of her will show:—"The best definition of a nurse," he records Miss Nightingale as remarking, "can be found, as always, in Shakespeare." It occurs in *Cymbeline*, where Lucius, in the fifth act, pleads that his "boy, a Briton born," may be ransomed. "Never master had," he exclaimed, "a page so kind, so duteous, diligent, so tender over his occasions, true, so feat, so nurse-like." As this page was really Imogen in disguise, it is a genuine tribute to the woman nurse. We say this lest male nurses should claim the implied compliment too hastily. Another point in Shakespeare of more medical interest was also singled out by Florence Nightingale for special praise. This was his treatment of death-beds. As Sir Edward Cook puts it: "In a characteristic appendix to her *Notes on Nursing*, Miss Nightingale discusses 'Some Errors in Novels,' pointing out, among other things, the untruth of death-bed scenes in works of fiction. "Shakespeare," she says, "is the only author who has ever touched on the subject with truth, and his truth is only on the side of art." The qualification is deserved; but is she really right in stating that he is the only author who ever held the mirror up to death correctly? What an original subject for a literary competition! We commend it to the *Westminster Gazette*; and, if it seizes the opportunity, trust we may be informed of the results arrived at.

### AN ILL-CONSIDERED REQUEST.

In a letter published in the *Times* and other papers, signed by the Bishops of London, Birmingham, and Durham, by ministers of several denominations, a few practitioners, some authors, journalists, and others, an appeal is made to all citizens to forego the use of meat and alcohol for at least one day every week, Thursday and Monday being suggested. It would have been better had each signatory stuck to his last. We gravely doubt the wisdom of the majority of members of either sex agreeing to this voluntary abstention, seeing that for the most part they are engaged to the full extent of their powers and opportunities upon war work, often of a very exacting and absorbing character, which means a larger output of energy from each than they have probably yielded heretofore in the course of their life. Human machinery, like all other, requires constant and intelligent attention. For ardent workers suddenly to change their diet and alter their habits whilst they continue their output of maximum energy is to invite a crisis which might compel cessation from work and even a breakdown in health. No doubt the appeal is well intentioned, but a certain place, which bishops at least may avoid, is reputed to be lined with these preludes to disasters and punishments which are wisely avoided by intelligent citizens. The business at the present moment of all citizens in this country is war, and economic causes must in the ordinary course regulate their dietary. This fact seems to have escaped the notice of the bishops and other signatories to an appeal that, would never have been drafted did it owe its origin to common-sense and not to sentiment. Appeals of this type, as *Truth* insists, do not, and properly, produce a wide response, nor will they, as we show elsewhere, effect any practical good.

### TEMPERAMENT AND FAMILY LIFE.

We desire to call the attention of our readers and the Press to the series of articles on Insanity and Temperament, the first of which appeared in the issue of The Hospital of May 6. We publish a second article in the present number. The truth is that there is no influence which makes so strongly for good or for evil, for unity in the family or for dissension, for misery or for happiness, for love or hatred, for life-long friendships or divisions, for hell or heaven on earth, for success or failure, for heroism or self-centred cruelty, for all that is best in life and all that is worst, than this question of temperament, especially in individuals who do not possess the power of personal discipline, and have never had the advantage of enforced discipline from their earliest years.

### RE-EXAMINATION OF REJECTED RECRUITS.

One of the serious defects of what compulsionists call the recruiting muddle, now remedied by the new Compulsion Bill, was the haphazard, inconclusive, and unsatisfactory way in which medical inspection was conducted. Under all the voluntary schemes, including that with which Lord Derby's name was associated, a man had either to be fit for active service abroad or else to be rejected as medically unfit. The evil results of this inevitable concomitant of the purely voluntary system were mainly two: first, many men were passed as fit who were not fit, and broke down soon or late under the strain of active service; secondly, many men were rejected who were quite capable of doing some essential service which would release a fitter man for the Front. In both directions there was waste of the national resources; and, further, there were undoubtedly men rejected who should never have been rejected at all. Under the new Bill every man rejected since August 14, 1915, must submit to re-examination, a waste of effort which could have been long since avoided if only the politicians could have made up their minds for themselves about the nation's necessities, instead of waiting until stern facts and the general voice of the people forced a decisive policy upon them.

### ALLOWANCES FOR WOUNDED SOLDIERS.

An Army Order was issued on May 6 announcing that a special temporary allowance shall be granted to a soldier discharged as "no longer fit for war service," for any period that may elapse between the date of such discharge and the decision of the Commissioners of Chelsea Hospital regarding his claim to a pension. The allowance will be paid by the Central Army Pension Issue Office, and will be recoverable from any pension or gratuity in lieu that may be awarded. The rate of the allowance is to be 20s. a week for men in respect of whom separation allowance, dependants' allowance, or family allowance was previously issuable; and half that amount for others. This reform is one which has been powerfully advocated by Sir Frederick Milner and others in the Press; it removes a source of great and indefensible hardship which has seriously affected many of the broken heroes of this war. It is a pity that some branches of the War Office seem only able to do the right thing after a popular outcry has been raised and the nation's conscience touched thereby. Perhaps we ought to be thankful that right does get done in the end, as a rule.

### THE VISUAL STANDARDS OF THE BRITISH ARMY.

Some searching criticisms of the standards of vision now in force in the medical examination of recruits for our Army are contained in a communication by Messrs. Paterson and Traquair, of the Edinburgh Royal Infirmary (*Lancet*, May 6, 1916, p. 954). Their argument is admirably concise and lucid, and supports certain definite conclusions. The present sight test of the British Army is, they find, the most severe of any in Europe, and excludes from the fighting-line large numbers of men who would be freely accepted as combatants in the armies of the Continental Powers. This is due to the fact that in the British sight test the uncorrected vision (without glasses) is thought more of than the corrected vision (with glasses); and a further mistake is our insistence on a certain standard of vision for both eyes, a less certain guarantee of real military efficiency than a higher standard of vision for one eye regardless of the condition of the other. Thus the writer of this note remembers an occasion on which he had to refuse a recruit who had lost the sight of one eye in an accident, but had splendid sight in the other eye; the man pointed out that he had been in the Territorials for years, and had repeatedly been a "marksman." Messrs. Paterson and Traquair conclude that there is urgent need of a remodelled sight test based upon corrected vision; common-sense and military experience alike support their view.

### GERMAN SURGICAL "STATISTICS."

A good deal of attention has recently been paid to the widely disseminated accounts of the second War Conference of German surgeons, held recently in Berlin. The information comes, of course, from enemy sources, and is therefore very highly tainted with suspicion. The statistics are, moreover, in some cases patently ridiculous: such as the repetition of previous assertions that 90 per cent. of the German wounded are able to return to active service, thanks to the superiority of German surgeons to those of all other nationalities. If no account be taken of deaths in the field hospitals and dressing stations, it is possible that 60 or even 70 per cent. of those wounded who reach Germany recover sufficiently to be made some use of, though many are doubtless incapable of field service; such tasks as sentry on railway lines, on neutral frontiers, guard over prisoners, and clerical occupations must necessarily absorb a large number of men, many of whom need not be very able-bodied. But these figures of 90 per cent. and over no medical officer with war experience can be got to regard as within the bounds of possibility. At the same time there is one factor which may possibly account for a higher percentage of recovery from rifle and machine-gun bullet wounds among the Germans than among the Allies: and that is the inferior smashing power of the French military bullet. Our own bullet, copied from a German model, is quite as deadly in its mutilating effects as that of the Huns: perhaps even a shade more horrible in its results. But the French bullet is a good deal less liable to break up and to deform its victims. It may be added that both the German bullet and the British one come within the limitations prescribed by the Hague Convention, in letter, at least, if not in spirit.

### THE CRAZE FOR GIFT SALES.

People like getting something for their money; and of all the things for which they are willing to pay a sensation is the one for which they will pay most. This instinct, of which betting (the chance of getting something for nothing) is the classic example, is a fundamental characteristic of mankind, and only absurd people inveigh against it; though, like every other human instinct, it is open to grave abuses. To this human characteristic is due in part the present popularity of the gift sale, a mode by which money is readily obtained from people who would otherwise remain deaf to the claims of the public work which is undertaken by the institutions that benefit by these proceedings. Institutional managers have not been slow to make use of this fashion. Its most curious feature is the way in which articles are purchased again and again, and so realise a very great deal more than their market value. Perhaps the pleasure comes as much from the fun of rival bidding as from seeing other people pay out! At a sale lately held at Arbroath with a view to the new infirmary buildings being opened free of debt, a cow, after passing from one purchaser to another, eventually realised £180. Of all the many modes of raising money for charities by giving the public a sensation, the gift sale is probably the least objectionable. How long will its popularity last?

### A DESERVING CHARITY.

There is a species of charity which proverbially begins at home; and there is a more altruistic type of that virtue which does not conform to such materialistic mammon worship. By no class is this latter sort so well displayed as by the theatrical profession, whose members are always ready to sacrifice their own ease and leisure in aid of charity, whether on the stage or off it. This well-known trait of generosity has been sometimes traded upon, and it has seldom been so frequently evoked or so bountifully displayed as has been the case since the outbreak of the present war. It is, therefore, fitting that when the "profession" does set out to help its own charities, the public should support the performance lavishly and cheerfully. In particular, the *Theatrical Garden Party* in aid of the Actors' Orphanage is deserving of a bumper attend ance; it is to be held, as usual, at the Botanic Gardens, Regent's Park, and the date selected is Tuesday, July 11.

### A BELATED SETTLEMENT.

At length Lancashire panel doctors' accounts are to be settled up to the end of 1914! The Panel Committee complained bitterly to the County Insurance Committee about the heavy arrears of fees, and a demand was made for a settlement of 1914 and 1915 accounts, and full debit and credit statements to be furnished every doctor on the panel from January 1914. In the event of both these demands being refused, the panel practitioners of Lancashire, said the communication, "will be compelled to reconsider their position." The Insurance Committee have meanwhile arranged to meet the accounts for 1914, the sum available for distribution being equal to 6s. 7.48d. per patient. For their 1915 bills the doctors will apparently have to "wait and see."

### AN EXTRA GRANT FOR WELSH UNIVERSITY EDUCATION.

A White Paper presented to the House of Commons on the subject of Welsh University Education contains some stringent criticisms of the failure of Wales to help in maintaining the constituent colleges, especially those of Bangor and Aberystwith. However much or little the people of Wales as a whole can be reproached in this respect, Cardiff and the medical school part of the University do not deserve any share of the blame. This seems to be admitted by the Treasury authorities, for they have felt justified (though with hesitation) in including in the estimates for 1916-17 an additional sum of £5,500 for the first year of the new grants (to the school). No condition as to supplementing this is attached during the continuance of the war; but when the war is over, an equal sum to be raised from the Cardiff rates will be insisted upon. Not only so, but up to a total maximum of £11,000 the Exchequer will grant an additional £500 for every extra £500 raised by the local authorities. Further still, half the additional annual cost of maintaining the school, up to a maximum grant of £5,000 a year, will be paid by the Exchequer. These proposals are combined with clear intimations that Whitehall does not mean to part with a controlling voice in the affairs of the University, for which there are doubtless many good reasons.

### THE SHADWELL HOSPITAL REPORT.

The forty-seventh annual report of the East London Hospital for Children, better known as "Shadwell," shows how even a children's hospital can help the military in these days of stress. The "Princess Mary" Convalescent Home at Bognor has not been open at all during the year 1915; but for six months it was taken over by the War Office for the billeting of troops. Apparently the War Office paid only £114 for this privilege; at least the only item of income in the accounts which could represent rent of the Home is for that amount. On the expenditure side of the accounts (of the Home) we note £51 for auctioneer's expenses in connection with "proposed sale of Convalescent Home." A reference to these proposals and their outcome would surely have been appropriate in the report of the board of management to the governors? The only other noticeable features in the accounts, which are most excellently kept and presented, are the entire absence of workmen's contributions to the hospital funds, and the drastic writing down of securities to their market values; the latter is an act of courage and common sense which might well be more generally imitated by boards of management. There is a debt to the bankers of £4,200, which the board are earnestly appealing to have cleared off.

### A GROSS ANACHRONISM.

When the late board of management of the Victoria Hospital, Blackpool, resigned some eighteen months ago, after a prolonged period of controversy, the idea was not only to change the *personnel* which had so long monopolised the institution, but to arrange matters in such a way that the old *régime* could not recur. In recording the plans for reform, which followed the resignation of the old board in August 1914 (when the management became vested in the trustees), The Hospital said: "The board is now to be reconstructed, and that means that a narrow clique . . . has been routed . . . The revision of the trust deed is to follow on democratic lines, which we hope will put an end once and for all to the voting powers previously absorbed by various companies and organisations.'' What has actually occurred? At a recent meeting of the hospital's subscribers it was stated that the trust deed had already been revised. From the chairman's remarks it also appears that the evil custom of cumulative voting still remains. As the *Blackpool Gazette* well says: "What is the use of getting rid of one set of persistent office-holders if they are only to be succeeded by another set, who may hang together for an even longer period?" If this anachronism is to be cynically perpetuated in this way, the Victoria Hospital will immediately forfeit its recent claims to public support, and the blow to its prestige, which is only too well remembered, thus repeated, may prove fatal to its reputation and usefulness.

### THE SMALL WARD IN SANATORIA.

Two important extension schemes, respectively finished and unfinished, are being undertaken at the Monsall Hospital, Manchester, and at Baguley Sanatorium. Both are municipal institutions. At Monsall five new blocks, containing eight twelve-bed wards, and an isolation unit have been added. When the beds in the observation wards attached to the large wards are included, a total of 112 beds has been added for the benefit of scarlet fever, diphtheria, and other infectious cases, including those which have been accommodated at Baguley. The result means that the staff is increased by thirty-six, and a new block has been added to the nurses' quarters. Baguley now becomes wholly a sanatorium, and by these changes its accommodation is increased by thirty-two more beds for women and 100 more for men. In each case, by the way, a series of small wards has been provided for them, accommodating one or two, and, in the case of the men, one, two, three, four, and six patients respectively. The small ward, except for single cases, has not, on the whole, stood well the test of experience with consumptive cases; and, though no one would suggest a return to the long double line of beds (after the infirmary type in passing days), the large, airy wards with six beds, such as may be beautifully seen at Northwood Sanatorium, are surely preferable to a collection of small rooms. In any case a two-bed ward is seldom, if ever, desirable for tubercular cases.

### THE WIDOW'S MITE.

A remarkable story was told by Mr. P. J. de Paravicini, as chairman of the King Edward VII. Hospital, Windsor, in announcing at a recent meeting a bequest of £1,000, which had been left by Mrs. Golden, at her husband's request, to the institution. Mr. Paravicini explained that this bequest represented the lady's husband's savings, which had been religiously left untouched by her. Mrs. Golden's own means were very slender. She lived in small rooms, and voluntarily went without coal in the winter, and scarcely allowed herself the necessaries of life. It was, he thought, a fine instance of self-sacrifice. The widow's mite in modern life has rarely been given more in the spirit of the old story than in the case of Mrs. Golden's devotion to an idea.

### THE CHILDREN'S COUNTRY HOLIDAY FUND.

The managers of this excellent charity have with praiseworthy enterprise enlisted the Chairman of the London County Council in support of their Fund. As head of the Education Authority for London, Mr. A. F. Buxton, who bears a name deservedly honoured in the annals of philanthropy and charity, is naturally in a position to realise the benefits which the Children's Country Holiday Fund confers; and his appeal is therefore certain of a respectful hearing. It appears from a letter which Mr. Buxton is issuing to the Press that war-time difficulties have hit this charity especially hard, not only through dire inertia of income but also by depriving it of many voluntary workers, now presumably helping their country's cause in other ways. The details of the admirable work which this Fund does are probably familiar to many of our readers; those who desire to help it or to know more of it may note that the offices are situated at 18 Buckingham Street, Strand.

### COURAGE IN PUBLIC HEALTH WORK.

The rarest of the virtues is courage, especially the moral courage required in civil life. A significant tribute to its value in a public official, holding the post of lady health visitor, was lately paid by a member of the Hawick School Board. Speaking of Miss Elliott, who has resigned from that post under the Board, the member remarked that not only was she an excellent public servant, but that the opposition which she had raised in some quarters was a proof of her thoroughness. By his recommendation that the Board should wait before making another appointment "until a medical officer for the schools should be found who would stand the attacks that she had had to endure," the lady health visitor received a very remarkable tribute. The Board agreed to adopt this suggestion—so Miss Elliott's work in the district still lives on in the influence which she has left behind her. No more gratifying result could be imagined to a courageous official.

### TROUBLE AT A NEW ZEALAND HOSPITAL.

It appears from the *New Zealand Medical Journal* that friction between the honorary medical staff and the board of the Timaru Hospital has recently come to a head. Some time back the board set up a committee to inquire into the method of keeping the store book, and the committee found the matron had been somewhat lax in making entries. The honorary staff seems to have interpreted this as a reflection on the matron's honesty. She, through her solicitor, asked for further inquiry; on this being refused the honorary staff and all the other medical men in the town signed a letter to the board demanding it. This also was refused; the honorary staff then resigned, and all other signatories of the letter. They then asked

Dr. Valintine, Inspector-General, to visit Timaru and conduct an inquiry. He has consented to this but cannot come before the end of the month; so the doctors wrote to the board asking leave to withhold their resignations in the meantime. The board has questioned the right of the Inspector-General to interfere, and by eight votes to three resolved to accept the resignation of the honorary staff, the resident surgeon to make the best arrangement he can for assistance at operations until the board can secure a permanent assistant resident surgeon (who may be allowed outside practice). In the course of the discussion some members of the board indicated that they had a grievance against the honorary staff and other outside doctors, in that they sent to the hospital no patient who could pay them, therefore patients' payments were a small item of the revenue. Particulars of the establishment, beds, and officers of this hospital are given on page 681 of *Burdett's Hospital Annual*, 1915.

### DEATHS BY POISONING.

The recently published annual report of the Registrar-General of Births, Deaths, and Marriages in England and Wales for 1914 shows that the number of deaths from poisoning and the kinds of poisons used are much the same as in previous years. Hydrochloric acid, oxalic acid, carbolic acid, and laudanum are the substances which accounted for the greatest number of cases of suicide, these four poisons alone being used in 280 cases. The reason for the selection of the first three, at any rate, is no doubt that they are readily available, but it is to be hoped that the increased restrictions that have been imposed on the sale of laudanum will have the effect of diminishing the number of deaths due to this poison, which also accounts for a considerable number of accidental deaths.

### THE ARMY COUNCIL AND WOMEN DISPENSERS

Some eight months ago the War Office began the practice of appointing women dispensers at military hospitals, the object being to release men, who had been engaged in the work of dispensing, for foreign service. Apparently the War Office is satisfied with the way the work has been done by women, notwithstanding that many of them, probably the majority, only possess the assistant's diploma of the Apothecaries' Society, for an Order has been issued by the Army Council which will effect a further substantial reduction in the number of male compounders employed. In future only those men are to be retained whose services, in the opinion of the commanding officer, are indispensable, and unless they pass this test even men over military age are to be released. It is not clear why the Order should be made to apply to men well over military age, unless it is desired that such men should be engaged to work as Insurance Act dispensers in civil pharmacies, in order that they may fill the gaps caused by young pharmaceutical assistants joining the Forces as combatants. By the way, the general employment of women holding only the Apothecaries' Hall assistants' qualification in military hospitals is likely to give rise to the interesting after-war problem: If women Apothecaries' Hall dispensers have been found good enough to compound medicines for our soldiers without being under the supervision of a pharmacist, are they not good enough to dispense medicines for insured persons and civil population generally?

### "PRINTER'S PIE, 1916."

In spite of the strains of war upon printers, publishers, editors, authors, and artists, this excellent publication will appear on May 15, and, judging by the list of contributors, it should be as bright and cheery a shillingsworth as it has always been in the past. How far it will reflect war topics, or avoid them, remains to be seen; there is something to be said for both methods of setting out to entertain people nowadays, and perhaps the editor will steer a middle course. But it is, in any event, a fairly safe prediction that to those on active service abroad, as well as to the sick and wounded in hospitals or elsewhere at home, it will prove a welcome alleviation of dull days or pain-racked nights. The philanthropic might do worse than order a dozen or two of copies for distribution in the nearest military or civilian hospital.

### THIS WEEK'S DRUG MARKET.

While on the whole the advancing tendency in the prices of drugs continues, the high values of some of the more commonly prescribed products are not so firmly maintained. Manufacturers of bromide have made a substantial reduction in their quotations. Salicylic acid and sodium salicylate are also obtainable at prices below the highest at which they have been offered; the reason for this is that British manufacturers have succeeded in increasing their output and improving its quality. Potassium permanganate is another article which has receded somewhat from the extremely high figure at which it has recently been sold; one of the reasons for this is that the fabulous prices have seriously curtailed the demand, and another that supplies from neutral countries are rather more freely available. Cocaine is also offered at prices below the maximum recently attained. The high prices of citric and tartaric acids are well maintained. Balsam copaiba is dearer. Digitalis leaves and senna leaves are fetching still higher prices. Liquorice root and arnica root continue very scarce. At the recent public sale of drugs in Mincing Lane there were few noteworthy alterations in price, but honey sold at higher rates and menthol was somewhat lower.

### TO OUR READERS.

**Contributions are specially invited from any of our readers to these columns. They should deal with topical subjects and news. They must be authenticated for the information of the Editor only. The minimum payment if published is 5s. There is no hard-and-fast rule as to space, but notes of about twenty lines in length are preferred.**

